# BSE infectivity survives burial for five years with only limited spread

**DOI:** 10.1007/s00705-019-04154-8

**Published:** 2019-02-24

**Authors:** Robert A. Somerville, Karen Fernie, Allister Smith, Keith Bishop, Ben C. Maddison, Kevin C. Gough, Nora Hunter

**Affiliations:** 1 The Roslin Institute and R(D)SVS, University of Edinburgh, Easter Bush, Midlothian, EH25 9RG Scotland, UK; 2 ADAS Biotechnology, School of Veterinary Medicine and Science, The University of Nottingham, College Rd., Sutton Bonington, Leicestershire, LE12 5RD UK; 3 School of Veterinary Medicine and Science, The University of Nottingham, College Rd., Sutton Bonington, Leicestershire, LE12 5RD UK

## Abstract

The carcasses of animals infected with bovine spongiform encephalopathy (BSE), scrapie or chronic wasting disease (CWD) that remain in the environment (exposed or buried) may continue to act as reservoirs of infectivity. We conducted two experiments under near-field conditions to investigate the survival and dissemination of BSE infectivity after burial in a clay or sandy soil. BSE infectivity was either contained within a bovine skull or buried as an uncontained bolus of BSE-infected brain. Throughout the five-year period of the experiment, BSE infectivity was recovered in similar amounts from heads exhumed annually from both types of soil. Very low levels of infectivity were detected in the soil immediately surrounding the heads, but not in samples remote from them. Similarly, there was no evidence of significant lateral movement of infectivity from the buried bolus over 4 years although there was a little vertical movement in both directions. However, bioassay analysis of limited numbers of samples of rain water that had drained through the bolus clay lysimeter indicated that infectivity was present in filtrates. sPMCA analysis also detected low levels of PrP^Sc^ in the filtrates up to 25 months following burial, raising the concern that leakage of infectivity into ground water could occur. We conclude that transmissible spongiform encephalopathy infectivity is likely to survive burial for long periods of time, but not to migrate far from the site of burial unless a vector or rain water drainage transports it. Risk assessments of contaminated sites should take these findings into account.

**Electronic supplementary material:**

The online version of this article (10.1007/s00705-019-04154-8) contains supplementary material, which is available to authorized users.

## Introduction

The epidemiology of the transmissible spongiform encephalopathies (TSEs, or prion diseases) varies substantially depending on the strain of TSE, and the species and *PRNP* genotype of the infected animals. Some scrapie outbreaks in sheep and goats appear to be spread both vertically from mother to offspring and laterally by physical contact and via environmental contamination [1]. Chronic wasting disease (CWD) is also thought to have a high degree of environmental spread [2–4]. By contrast, bovine spongiform encephalopathy (BSE) has shown little, if any-direct animal-to-animal transmission or environmental spread. Most cases can be ascribed to the consumption of contaminated feed [5] although BSE continues to occur sporadically, with less certainty of its route of infection. In Great Britain, the two most recent cases of BSE were reported from active surveillance of fallen stock in 2015 [Defra active TSE surveillance in Great Britain statistics]. The transmission of BSE to humans as variant Creutzfeldt Jakob disease (vCJD) is also thought to have occurred predominantly through consumption of infected material from cattle, although four human-to-human transmissions of vCJD are likely to have occurred via the use of blood products [6–8].

A hallmark of TSEs is an abnormal protease-resistant form (PrP^Sc^) of a cellular protein, PrP^c^ or prion protein. PrP^Sc^ is usually, but not always, associated with infectivity, and the conversion by PrP^Sc^ of PrP^C^ to form more PrP^Sc^ is so central to the infectious process that it is thought by most to be the only means by which these unusual infectious agents replicate. However, the link between PrP^Sc^ and infectivity is not absolute [9–11], and in some studies, including this one, it is important also to demonstrate the ability to initiate infection and cause clinical disease.

The high resistance of TSEs to inactivation has been recognised [12], so their survival in the environment is unsurprising. To test the extent to which TSE infectivity could survive, an experimental source of TSE infectivity (263 K scrapie) was buried with soil in a petri dish and exhumed after three years, after which the sample was still infectious and able to cause disease in hamsters [13]. More recently, 263 K retained the ability to infect hamsters after burial in sandy loam soil for 29 months [14]. Under small-scale laboratory conditions, TSE infectivity and PrP^Sc^ have been shown to bind to soil components, survive for long periods and migrate only short distances in soil columns [15, 16].

The binding and survival of TSE agents is influenced by soil composition and the TSE strain, with ovine scrapie being more persistent in some soil types than cattle BSE [17, 18], and oral infection may be promoted by binding of TSE agents to soil components [14, 19, 20]. There is also evidence that PrP^Sc^ can be taken up by plants from experimentally infected soil [21]. However, these and other experiments may not reflect the field situation when affected animal carcasses are buried for long periods of time.

A major outbreak of BSE in the UK with peak in incidence in the 1980s and 1990s is very likely to have led to infected animals and other materials ending up in the environment, for example, from burial of infected carcasses, deposition in landfill or waste from abattoirs. These sources could act as a reservoir of infectivity if cattle or other susceptible animals were to be exposed to these sources. The spread of BSE into the human population in the form of vCJD raises additional concerns for human health. The cull of large numbers of cattle arising from the UK foot-and-mouth disease (FMD) epizootic in 2001 resulted in a large disposal problem. In this emergency situation, carcasses were buried or burnt on funeral pyres (where they may not have been sufficiently consumed to destroy BSE infectivity) and disposed to landfill [22]. BSE incidence was reducing by 2001, but there were ~ 1,000 confirmed clinical cases, and a proportion of the animals slaughtered to control FMD are very likely to have been incubating BSE. BSE infectivity from buried carcasses may continue to survive for long periods of time in the environment and remain a potential risk for both livestock and humans.

We report here the results of field studies in which mouse-passaged BSE was buried either as a spike placed within cattle heads or as an uncontained bolus. Two types of soil (sandy and clay) were used in large containers exposed to the elements. Survival of infectivity was investigated over a five-year period by mouse bioassay of the surviving original inocula, of the surrounding soil and of the rainwater, which was collected after it had filtered through the soils.

## Materials and methods

### Experimental site design

**Fig. 1:**
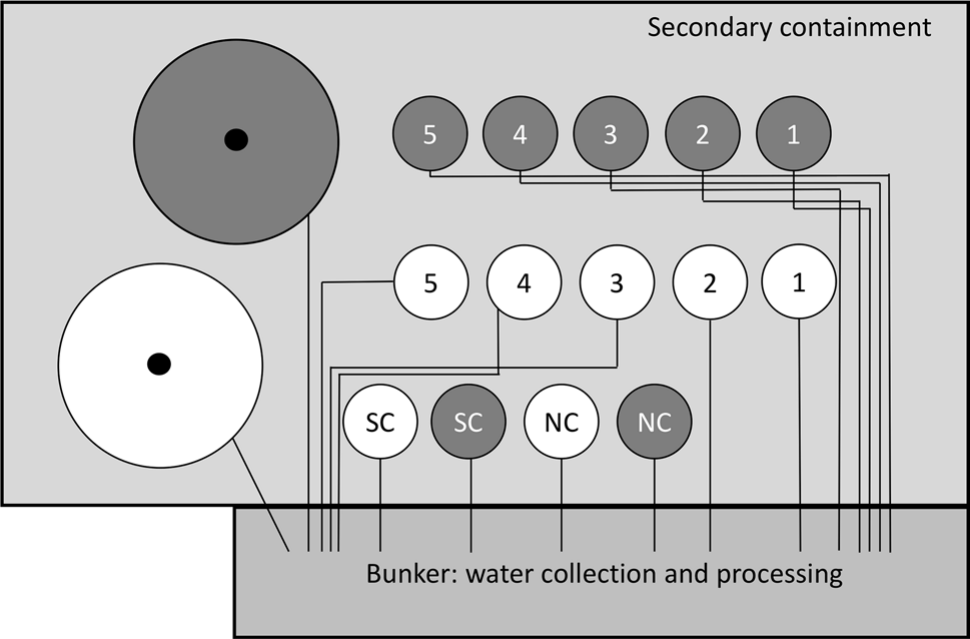
Schematic diagram of the field site and buried TSE infectivity. The completely enclosed site area (14 m × 7 m) held 2 large and 14 small circular lysimeters within secondary containment with a waterproof lining. Cattle heads were interred in numbered and ‘NC’ lysimeters; black circles indicate lysimeters containing boluses. Lysimeters were filled with clay (dark grey) or sand (white). Numbers 1-5 refer to the year in which each 301V-spiked head was exhumed. NC, negative control heads; SC, soil control (lysimeters contained no heads or bolus). Black lines indicate water drainage from each lysimeter to the bunker for filtration, collection and processing. The system also included an overflow tank required to collect water after heavy rain

The site, in the UK in eastern Scotland, was roughly 14 m long by 7 m wide and enclosed 14 small and two large circular lysimeters placed in secondary containment lined with a waterproof liner, which allowed nothing to spread or leak to the surrounding ground (Fig. 1). The small lysimeters were 1 m in diameter and 1.5 m deep, with a conical bottom to facilitate drainage. The large lysimeters were similar in design except that they were 3 m in diameter and 1.5 m deep. All lysimeters were filled by hand to within 30 cm of the top with either a clay or sandy soil provided by Brown Soil, UK, and the Macaulay Land Use Research Institute, UK. The physicochemical characterstics (analysis carried out by INRA, France) of the soils are given in Supplementary Table 1. The lysimeters were then layered with 50% each of subsoil and topsoil and then were allowed to settle for six months, bringing the surface back to ~ 30 cm level, prior to burial of bovine heads. The lysimeters were covered with a grill to prevent animal ingress but allow rainwater through and other environmental influences on the soil surface to occur. Drainage pipes from the bottom of each lysimeter were connected to collection vessels in the bunker (Fig. 1) to allow both the analysis of filtrates of the rainwater that passed through them and its subsequent decontamination and disposal.

Environmental conditions were monitored electronically within the site during the study. External air temperature and rainfall amounts were also recorded (Supplementary Figure S1). Prior to collection and decontamination, water draining from the lysimeters was passed through glass fibre filters (Millipore AP20 filters, Swinnex, 47 mm). Filters readily became blocked with small particulates that co-eluted from the lysimeters, after which the filters were automatically bypassed. The filters were collected and replaced approximately monthly throughout. Collected filters were stored at -20 °C.

### Burial of TSE-spiked bovine heads and bolus

Twelve bovine heads obtained from a local slaughterhouse were from animals less than 30 months old, passed fit for human consumption and presumed therefore to be free from BSE infection. The heads included the brain still *in situ* and the three top vertebrae of the spinal column. Ten heads were each spiked with 10 g of brain macerate from mice clinically affected with TSE, by injection via the bolt hole in the skull created when the cattle were culled. We used a mouse-passaged model of TSE, the 301V strain, which was derived from BSE by serial passage in VM mice, since this allowed known amounts of infectivity at high titre to be used to spike the experiment and sensitive detection by assay in mice with relatively short incubation periods [23]. Two heads were not spiked and served as negative controls (NC). The heads were buried, one in each of 12 small lysimeters (6 clay and 6 sandy soils), in holes dug such that the soil level, and therefore the bottom of each head, was 0.5 m from the surface, all with the same orientiation, arbitrarily chosen, lying horizontally with the top of the head to the south and noses pointing east. As positive controls, individual 301V-infected VM mouse brains were placed in plastic bijou bottles and buried at the nose of each cow head, to be recovered at exhumation. Two further lysimeters (one each of clay and sandy soils) had no cattle heads buried and served as negative soil controls (SC) for the whole project. In a complementary study, 100 g of 301V-infected mouse brain macerate was frozen and then buried, as an unprotected bolus, at 0.5 m below the surface of each of the two large lysimeters, one each of clay and sandy soil.

### Sampling procedures and infectivity assays

One head from a small clay lysimeter and a small sand lysimeter were exhumed after one year in the ground, and this was then repeated annually with the other heads for up to 5 years. Prior to exhumation, soil samples were collected from directly above the heads (central, Cen) and at points north (N), south (S), east (E) and west (W), each 25 cm horizontally from the middle core, using a 3-cm diameter corer until the skull was reached or to a depth of 50 cm. The cores were divided into sections of approximately 10 cm each in length (i.e., a sample labelled 10 cm depth represents soil sampled between 0-10 cm, etc.), which were then mixed, aliquoted and frozen at -20 °C. The head was then removed, and further soil samples were taken at 60, 70, 80 and 100 cm depth in N, S, E and W directions and directly below the burial point (Cen). Soil samples were stored frozen at -20 °C.

Heads were dissected and brain cavity contents recovered. They were divided into six regions, in as much as the residual brain structure allowed, and two 1-*g* samples were taken from each region and frozen at - 20°C until processing. To each 1-*g* sample, 1 ml extraction solution was added (1% sarkosyl, 100 mM sodium phosphate, pH 7.4, containing 40 µg of proteinase K [PK] per ml) and then incubated at 37 °C with shaking for one hour. PMSF was added (20 µl, 100 mM) followed by centrifugation at 1,000 *g* for 10 minutes. Two 320-µl volumes from each aliquot were added each to 1180 µl of methanol and incubated at -20 °C overnight. After centrifugation at ~ 13,000 *g* for 10 minutes, the methanol was removed and the pellet dried. Each pellet was resuspended in 100 µl of 0.9% saline, and the two extracts were pooled.

For soil samples, the same sample-processing procedure was followed for 1 g of soil as detailed for brain. For clay soil, these extracts were used for inoculation into mice to confirm infectivity levels following a final centrifugation step (2,000 *g*) to clarify the solutions. Extracts from sandy soil required further treatment to ensure no very fine particulate matter remained in suspension. Just prior to inoculation and during the clarifying centrifugation step, a cushion of 100 µl of 20% (w/v) sucrose was carefully layered at the foot of each tube so that any pelleted material would remain undisturbed when the supernatant was withdrawn for injection. Groups of four VM mice per sample were then inoculated intracerebrally with 20 µl of each extract.

Positive controls consisted of individual 301V-infected VM mouse brains that were placed in bijou bottles, buried at the nose of each cow head, and then recovered at exhumation. These samples were inoculated in parallel with each of the soil samples each year as a control for the bioassay. For this, 10 µl of 10 % (w/v) mouse brain homogenate was intracerebrally injected into each of four VM mice per group. In addition, extraction controls were carried out each year by spiking samples of each soil with 301V brain extracts and carrying out the same extraction procedure as for the lysimeter soil samples as follows. Briefly, 1.2 g of the appropriate soil was added to 400 µl of water, after which 10 µl of 10 % (w/v) of the pooled 301V brain homogenate was added and the same extraction method was used for clay and sand as described. Negative controls were taken from buried uninfected cattle heads (NC lysimeters) and from a separate pair of lysimeters without buried heads (SC lysimeters) and treated exactly as for the spiked infected samples.

Vertical core samples, taken using the same method for the small lysimeters, were also collected from the large lysimeters at 25 and 50 cm from the centrally buried bolus at various times up to 48 months after burial and on a different radius each time; depths of 40 to 70 cm were taken. The resulting holes were backfilled with clay or sandy soil reserved for the purpose. Most samples were stored for future analysis and are not analysed here. At the end of the experiment, a central core was taken using a 16-cm diameter core with ~ 1.2 m length, ensuring that the site at which the boluses had been placed was sampled and stored as above. Each soil sample (1 g) that was analysed was processed and used to inoculate groups of four VM mice.

A limited bioassay was carried out to assess levels of infectivity in the filters used to collect particulate samples from rainwater eluted from sand and clay lysimeters. Four filters were assayed, two each from clay and sandy-soil lysimeters and removed from the elution lines at 4 and 25 months after the burials. For analysis, 5 ml of extraction buffer was added to each sample (1% sarkosyl, 100 mM sodium phosphate, pH 7.4, containing 40 µg of PK per ml) and rotated overnight at room temperature. Four VM mice were inoculated intracerebrally with 20 µl of each extract.

Incubation periods were recorded as the time between inoculation of the VM mice and the development of clinical signs, confirmed by the presence of vacuolation in brain sections (H&E-stained) [24]. Animals were culled at the development of clinical signs or at the termination of the experiment at ~ 700 days. All use of animals, the collection of animal tissues, and the use of such tissue were carried out in accordance with the Animal (Scientific Procedures) Act (ASPA) 1986, under licences from the UK Government Home Office (Project Licence 60/2544). All animal experiments were subject to review and approval (01-124) by The Roslin Institute Ethical Review Committee, and euthanasia methods were approved by the UK Home Office.

### Detection of PrP^Sc^ within filter extracts

Detection of PrP^Sc^ from soil samples was done using a previously developed method [16], in which sarkosyl and proteinase K (PK) were used for the extraction of PrP^Sc^ from the two soil types and extracted murine PrP^Sc^ was analysed by western blot using the anti-PrP antibody 6H4 [25]. Due to the difficulty of successfully extracting small amounts of PrP^Sc^ from large volumes of soil, the results obtained by this method were not useful and are not presented here.

A serial protein misfolding cyclic amplification (sPMCA) technique was used to analyse filter extracts taken across the lifetime of the 4-year experiment. Optimisation of the 301V BSE sPMCA used in this study has been published elsewhere [26]. Briefly, the amplification conditions comprised a 10 % (w/v) VM mouse brain homogenate substrate consisting of 20-40 murine brains homogenised at a time by bead beating. sPMCA reactions were carried out in duplicate in 200-µl thin-wall PCR tubes containing 90 µl of brain homogenate substrate, 5 µg of digitonin, three 2.4-mm Teflon beads and 10 µl of the sample to be amplified. Reaction tubes were placed in a Misonix S4000 sonicating water bath set on a program of 10 seconds of sonication every 30 min for 24 hours at a power setting of 190-200 W at 37 °C. Every 24 hours, samples were diluted tenfold with fresh VM brain substrate and sonicated for a further 24-hour round of repeated sonication and incubation. Amplifications were carried out for five days, after which reaction products were digested with PK at a concentration of 100 µg/ml for 90 minutes at 40 °C. Digested samples were screened by boiling for 5 minutes in 1xLDS buffer (Invitrogen), spotting onto nitrocellulose filters, blocking for 1 hour with 3 % (w/v) skimmed milk and probing with the anti-PrP antibody SHa31 [27]. Bound antibody was visualised using a HRP conjugate and chemiluminescent substrate. All samples that showed at least a single positive test sample in an initial screen were then re-amplified a second time with the same controls to confirm the results (all results correlated upon this repeat analysis). Positive dot blot samples were confirmed by western blot: samples were digested and denatured as for dot blotting and electrophoresed through a NuPAGE SDS-PAGE gel system (Invitrogen). Proteins were then transferred to a polyvinylidene difluoride membrane by electroblotting, and the membranes were blocked and probed for PrP^Sc^ as described for dot blots. Test and control samples were provided and analysed blind before being decoded. Blinded controls, consisting of spiked filter extract samples (samples with known 301V titres) and negative controls (unspiked filter extracts) were included in each sPMCA experiment. In addition, known positive (filter extract from the SC lysimeter and spiked with 301V) and negative (filter extract from the SC lysimeter) controls were included in the analyses, and the filter sample taken at month 25, which was positive by mouse bioassay, was also analysed by sPMCA.

## Results

### Survival of 301V in buried cattle heads

To model key aspects of the environmental fate of TSE infectivity with respect to scale, time, and natural weather and soil conditions, we set up two field experiments to test the long-term survival of TSE infectivity under near-natural conditions. In the first experiment, ten cattle heads were spiked with 10 g of a previously titred preparation of 301V-infected mouse brain (10^7.7^ intracerebral ID_50_/g in VM mice with incubation periods ~ 120-140 days for dilutions with 100% attack rates, 10^−1^-10^−4^) [28] and buried in lysimeters containing either a clay soil or a sandy soil, selected to provide contrasting properties of composition. Details of the soil characteristics are presented in Supplementary Table 1. The clay soil was fine textured with small pore spaces, and high water-holding capacity combined with slow drainage and high organic matter content. In contrast, the sandy soil had coarse texture, large pore spaces, low water-holding capacity with rapid drainage and low organic matter content. Various plants self-seeded on to the top of the lysimeters, and this vegetation was periodically cut back each summer.

**Fig. 2:**
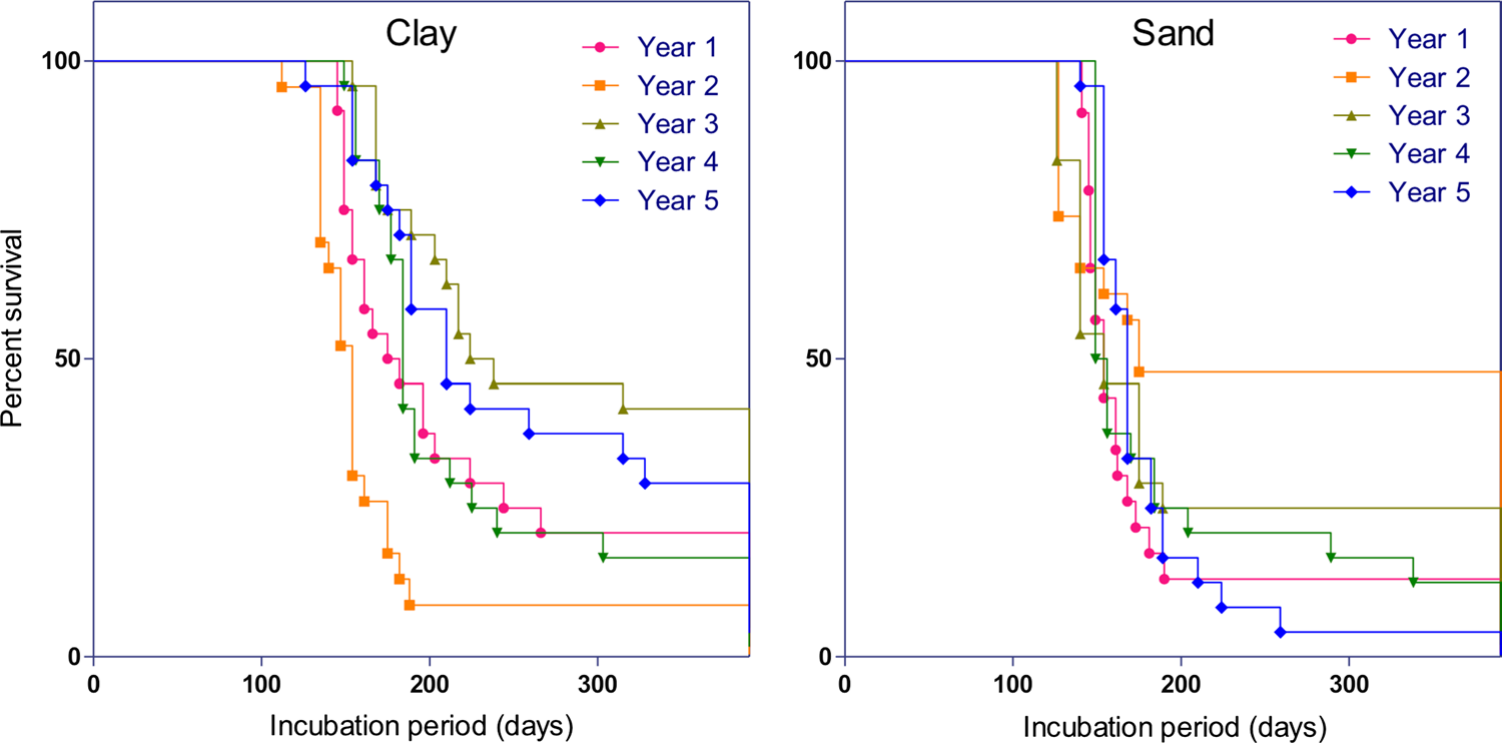
Survival of TSE infectivity in buried heads. The presence of infectivity was assessed by mouse bioassay. Percentage survival times (days) of mice from annual sampling of 301V-spiked cattle heads in clay and sandy soils are shown. Data for six brain regions (A-F) are pooled for each year

One bovine head from each soil type was exhumed annually. After two years, there was some liquefaction of the brain contents within the heads buried in clay soil. The contents of the brain cavity and some surrounding soil samples were tested for TSE infectivity by bioassay in VM mice (Table 1) [16]. Measurement of the incubation periods gives an approximate measure of the amounts of infectivity, since incubation periods are inversely related to titre. Six samples (here marked A-F) were analysed from each brain cavity each year, giving a total of 60 sets of bioassays. The results show that infectivity was detected in 57 of the samples (Table 1), indicating that this murine-passaged BSE was well dispersed within the cattle brain and that infectivity was retained over the 5-year period. Three bioassays gave no TSE-positive mice: one from each of two samples taken from the sandy soil at years 2 and 3 and one from clay soil at year 3. However, the other five samples in each of these heads were positive. Incubation periods in the mice ranged from a mean of 130 days up to a mean of 328 days. There was considerable overlap in incubation period length between mice inoculated with the two types of soil, and there was no clear difference between them (Fig. 2). These results suggest that infectivity survived at similar levels in all heads for the full duration of the study.

**Table 1:** TSE infectivity detected within bovine heads and in surrounding soil

		Clay												Sand											
	Year	1		2		3		4		5		SC		1		2		3		4		5		SC	
		N	IP	N	IP	N	IP	N	IP	N	IP	N	IP	N	IP	N	IP	N	IP	N	IP	N	IP	N	IP
		Samples taken from within the heads																							
*In head*	A	**4/4**	**149**	**4/4**	**165**	**3/4**	**201**	**4/4**	**167**	**4/4**	**173**			**1/4**	**181**	0/4		**4/4**	**137**	**2/4**	**237**	**4/4**	**180**		
	B	**4/4**	**155**	**4/4**	**134**	**2/4**	**193**	**2/4**	**174**	**1/4**	**175**			**4/4**	**153**	**4/4**	**130**	**4/4**	**130**	**4/4**	**149**	**4/4**	**173**		
	C	**4/4**	**162**	**4/4**	**151**	**4/4**	**165**	**4/4**	**184**	**1/4**	**328**			**4/4**	**158**	**4/4**	**130**	**3/4**	**180**	**4/4**	**179**	**4/4**	**168**		
	D	**3/4**	**222**	**3/3**	**144**	**4/4**	**214**	**4/4**	**207**	**4/4**	**205**			**4/4**	**161**	**4/4**	**130**	0/4		**4/4**	**202**	**4/4**	**165**		
	E	**2/4**	**186**	**2/4**	**185**	0/4		**4/4**	**198**	**4/4**	**186**			**4/4**	**143**	**3/4**	**268**	**3/4**	**156**	**4/4**	**151**	**3/4**	**201**		
	F	**2/4**	**234**	**4/4**	**140**	**1/4**	**315**	**2/4**	**205**	**3/4**	**226**			**3/3**	**160**	**2/4**	**235**	**4/4**	**152**	**4/4**	**153**	**4/4**	**159**		

		Clay												Sand											
	Depth (cm)	N	IP	N	IP	N	IP	N	IP	N	IP	N	IP	N	IP	N	IP	N	IP	N	IP	N	IP	N	IP
		Samples taken from the soil around the heads																							
N	40									0/3												0/4			
	50	0/4		0/4		0/4		0/4		**1/4**	**210**			0/4		0/1		0/4		0/4		0/4			
	60	0/4		0/4		**1/4**	**251**	**4/4**	**237**	**3/4**	**296**	0/4		**1/4**	**359**	0/6		0/4		0/4		0/4		0/4	
	70	0/4		0/4		**3/4**	**237**	0/4		0/4				0/4		0/5		0/4		**1/4**	**236**	0/4			
	80									0/4												0/4			
E	40									0/4															
	50	0/3		0/4		**1/4**	**H**	0/4		0/4				0/4		0/5		0/4		0/4		0/4			
	60	0/4		**1/4**	**307**	**3/4**	**H**	**2/2**	**181**	0/4		0/4		0/4		0/4		0/4		0/4		0/4		0/4	
	70	0/3		0/4		**3/4**	**239**	**4/4**	**188**	0/4				0/7		**1/3**	**H**	0/4		0/4		0/4			
	80									0/4												**1/4**	**295**		
S	40									0/4												0/4			
	50	0/3		0/8		0/4		0/4		0/4				0/4		0/4		0/4		0/4		0/4			
	60	0/4		**1/4**	**230**	0/4		**1/4**	**355**	0/4		0/4		**1/3**	**218**	0/6		0/4		0/4		0/4		0/4	
	70	0/4		0/4		**2/4**	**223**	**1/4**	**321**	0/4				0/4		0/4		0/4		0/4		0/4			
	80									0/4												0/4			
W	40									0/4												0/4			
	50	0/3		0/4		0/4		**4/4**	**283**	0/4				0/4		0/4		0/4		0/4		**1/4**	**295**		
	60	**1/3**	**183**	0/4		0/4		**4/4**	**129**	0/4		0/4		**1/4**	**317**	0/4		0/4		0/4		0/4		0/4	
	70	0/3		0/4		0/4		**2/4**	**254**	**2/4**	**172**			0/3		0/4		0/4		0/4		0/4			
	80									0/4												0/4			
Cen	60	**2/3**	**257**	ND		**4/4**	**267**	**4/4**	**169**	**2/3**	**252**	0/4		0/4		0/4		0/4		0/4		**1/4**	**204**	0/4	
	70	**1/4**	**218**	**1/4**	**H**	**4/4**	**190**	0/4		0/4		0/4		0/4		0/4		0/4		0/4		**1/4**	**267**	0/4	
	80	**1/4**	**239**	0/4		**2/4**	**174**	**3/4**	**244**	**1/4**	**203**			0/3		0/4		**1/4**	**H**	0/4		**3/4**	**255**		
	100									0/4												**2/4**	**288**		

Six samples from the skull cavity (A-F) were analysed along with soil samples taken from a central core above and below the head (Cen) and from cores 25 cm from the central core in N, E, S and W directions (soil depths of sample collection are also indicated). Samples were tested for TSE infectivity in mice. The number of TSE-positive mice per group/total number injected and surviving for assessment are shown (column N), with the average incubation period in days postinfection of each TSE-positive group (column IP). Those that were diagnosed TSE positive only from histological examination of the mouse brains after culling are indicated (H). Samples with any sign of infectivity are in bold. Soil controls (SC) were taken each year from lysimeters filled with sand or clay without cattle heads at the depths indicated

Negative controls taken from buried uninfected cattle heads (NC lysimeters) were all negative (0/20 at year 5). Buried positive controls (301V-infected VM mouse brains buried in bijou bottles) were analysed each year, and all samples were positive in all mice (4 mice per year for 5 years for lysimeters containing sand and clay, average incubation periods for each group of mice between 104 and 126 days). Extraction controls (301V-spiked soil) were also positive in all mice (3 or 4 mice per year for each of 5 years for lysimeters containing sand and clay, average incubation periods for each group of mice between 104 and 200 days).

Samples taken from the soils around the heads were also tested for infectivity that might have leaked from the infected brain cavities (Table 1 and Fig. S2). Overall, in the clay soil, 20 out of 68 samples tested contained TSE infectivity, most samples had incomplete attack rates, and the average incubation periods were from 129 to 307 days. For sandy soil, 7 out of 67 samples were positive, all with only single mice being affected and incubation periods between 218 and 359 days, and some mice were only positive by IHC examination upon culling. Infectivity was detected 25 cm lateral to the burial site in all years except year 3 with the sandy soil. For samples taken from underneath the head (depth ≥ 60 cm), the clay soil produced more positive samples compared to the sandy soil: 11 out of 15 positive samples, vs. 5 out of 16 positive samples. Also, for the sandy soil, 4 out of the 5 samples with TSE infectivity were in the year 5 sample. Samples at a depth of 60 cm from N, E, S and W direction and 60 and 70 cm for a central core were all taken from the soil control lysimeters (SC), and all samples were negative in groups of four mice (Table 1).

### Survival of 301V macerate in soils

A 100-g bolus of 301V-infected brain macerate was frozen and then buried 0.5 m below the surface in each of two large lysimeters containing either clay or sandy soil. The soil was sampled by taking a series of vertical cores of soil at horizontal distances of 25 and 50 cm from the bolus position at various times from 1 to 48 months after burial. Analogous samples from the two lysimeters were tested for infectivity by bioassay (Table 2 shows data for the lysimeter containing sandy soil). No TSE infectivity was detected in any of the samples assayed from these cores except for one soil extract from a core taken from the sandy soil after 12 months and 25 cm from the bolus and 60 cm depth, which caused TSE infection in a single mouse. Therefore, no further outlying samples were tested by bioassay.

**Table 2:** Summary of mouse bioassays of non-central core soil samples taken from the lysimeter containing sandy soil with buried boluses of 301V brain homogenate

Depth (cm)		25 cm distance from central core								
	Month:	1	3	7	9	12	18	14	36	48
40		X	X	X	X	0/4	0/4	0/4	0/4	0/4
50		0/3^#^	0/4	0/4	0/4	0/4	0/4	0/4	0/4	0/4
60		0/4	0/4	0/4	0/4	**1/4**	0/4	0/4	0/4	0/4
70		0/4	0/4	0/4	0/4	0/4	0/4	0/4	0/4	0/4

Depth (cm)		50 cm distance from central core								
	Month:	1	3	7	9	12	18	14	36	48
40		X	X	X	X	X	X	X	X	X
50		X	X	X	X	0/4	0/4	0/4	0/4	0/4
60		0/4	0/4	0/4	0/4	0/4	0/4	0/4	0/4	0/4
70		X	X	X	X	0/4	0/4	0/4	0/4	0/4

Samples were taken at 25 cm and 50 cm from central cores at different depths as indicated. Samples were taken repeatedly from 1 to 48 months and on four radii around the central core. X = not tested, samples stored (N.B. samples at 10, 20, 30, 80, 90 and 100 cm depths were also taken and stored). Each sample that was tested was inoculated into four mice (^#^1 mouse died of intercurrent disease), and results are presented as the number of TSE-positive animals/total injected. Samples with any sign of infectivity are in bold. The bolus level in the central core was 50 cm

After four years, a large core was taken vertically through the centre of each lysimeter so that the original locations of the boluses were sampled. In these samples (at 50 cm depth) in both clay and sand lysimeters, infectivity was detected (Table 3). Each sample caused TSE disease in all four of the mice inoculated and with incubation period means ranging from 153 to 224 days in sandy soil from and 135 to 165 days in clay. In addition, infectivity was detected in core samples below the site of the bolus from the sand lysimeter at depths from the surface of 60 and 80 cm (10 cm and 30 cm below the bolus), but fewer mice were affected, indicative of lower levels of infectivity and with incubation period means of 174-224 days. For the clay soil, a single mouse was TSE affected (incubation period 196 days) from one of three samples at 60 cm depth. However, unlike sandy soil, the clay soil above the bolus did test positive for infectivity. At 40 cm depth (10 cm above the bolus) all four inoculated mice developed TSE (with a mean incubation period of 173 days), and at 30 cm (20 cm above the bolus), 50% of the group was affected (mean incubation period 175 days). This difference may be due to the contrasting drainage properties of the two lysimeters. The sandy soil was much more freely draining, and hence, rainfall flowed through it relatively quickly. The clay lysimeter drained very poorly and often flooded after heavy rainfall. This was reflected in a much higher water table in the clay lysimeter most of the time, which may have promoted upward migration of infectivity.

**Table 3:** Summary of mouse bioassays of central core soil samples taken from lysimeters with buried boluses of 301V brain homogenate

Depth (cm)	Number of samples tested	Number of TSE-positive mice/number inoculated	
		Clay core	Sandy core
10	1	0/4	0/4
20	1	0/4	0/4
30	1	**2/4**	0/4
40	1	**4/4**	0/4
50	5	**20/20**	**20/20**
60	3	**1/12**	**6/12**
70	1	0/4	0/4
80	1	0/4	**1/4**
90	1	0/4	0/4
100	1	0/4	0/4
110	1	0/4	0/4
120	1	0/4	0/4

Samples were taken after burial for 48 months. Each sample was inoculated into four mice, and results are presented as the number of TSE-positive animals/total injected. Samples with any sign of infectivity are in bold. The bolus level was 50 cm

### Detection of 301V in eluted water from lysimeters

Amounts of water eluting through the lysimeter were very high. For example, 10 mm of rain resulted in about 70 litres of water falling on the large lysimeters and 8 litres on the small lysimeters. Details of rainfall over the course of the study are given in Supplementary Figures S1 and S3. In summary, recorded rainfall was ~ 790 mm in the first 12 months of the project and ~ 850 mm in the second 12 months. It is difficult to be certain how much eluent passed through the filters, as they were bypassed automatically if they became blocked by small particulates.

**Fig. 3:**
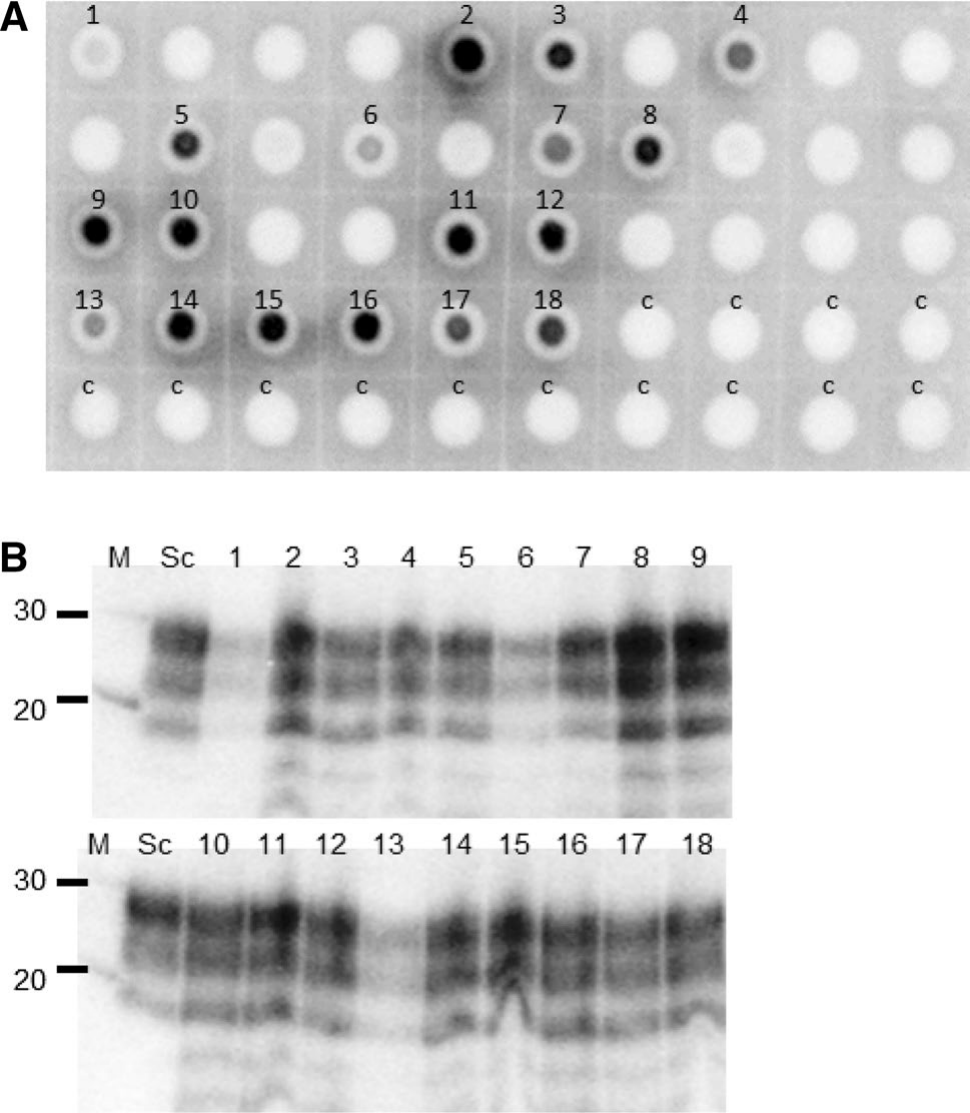
Representative immunoblot analysis of sPMCA products. Detection of PrP^Sc^ in filtrate was carried out by sPMCA. The products were first analysed by dot blot (A), and samples showing dot blot positivity were then confirmed as containing PrP^Sc^ by western blot (B). All samples were digested with PK before analysis, and the blots were probed with the anti-PrP antibody SHa31. sPMCA-positive samples are indicated (1-18). These include blinded control samples (2, 3), non-blinded control samples (9-12), and samples from filters removed at the following times (months) after bolus burial: 2m (5, 8), 3m (13,14), 4m (4), 6m (6), 7m (15, 16), 8m (1), 17m (7) or 25m (17, 18). For dot blotting, negative-control sPMCA reactions amplifying extract from two different control column water filter extracts are indicated (c; 7 replicate analyses for each extract). For western blotting, molecular mass markers (M) at 20 and 30 kDa are shown. Sc, PK-digested scrapie-positive brain sample as a western-blot-positive control

A limited number of filter extracts were analysed by bioassay. Four filter extracts were tested, two for each of the large lysimeters, taken at months 4 and 25. The 25-month filter sample from the clay lysimeter produced clinical disease in a single VM mouse with an incubation period of 182 days. In order to further investigate the filters, we developed an sPMCA method [26] and focussed on testing the filters from the large clay lysimeter. Samples were blinded and the results subsequently decoded by staff who had had no involvement in the sPMCA analysis. At least two aliquots from each sample were analysed, and the replicates were also blinded. Examples of the positive and negative dot blots of amplified products from the analysis are shown in Figure 3A, which is in a randomised order due to the blinding and includes positive control samples both known and unknown to those carrying out the analysis. In addition, amplified products from the positive samples were subjected to western blot analysis after PK digestion, and all show the typical band pattern for PrP^Sc^ (Fig. 3B). Seven out of the eight samples taken in the first 8 months of the project were positive in at least one instance by sPMCA, and a sample taken at 17 months was also positive. This included the filter extract that showed negative bioassay results (0/4) at 4 months, which was positive by the sPMCA method in one of the two replicate tests (Fig. 3, sample 4). In addition, the extract taken from the 25-month filter, which had been positive by bioassay, was also positive by sPMCA (2 out of 4 replicates tested, Fig. 3, samples 17 and 18). All sPMCA results (positive or negative) are shown (Supplementary Fig. S3). The negative controls analysed (one blinded sample and two non-blinded samples that were analysed 16 times in total) did not amplify any PrP^Sc^.

## Discussion

The resistance of TSE infectivity to inactivation is well documented [12, 29], as is the apparent long-term survival of scrapie on pastures [30]. Therefore, the question of disposal of carcasses of BSE-affected cattle is of considerable concern in the UK. Burial of farm animals that are found dead has been prohibited by the EU Animal By-Products Regulations since May 2002. However, there are exemptions to the rules. If animals die in remote locations where transport to processing plants is difficult or impossible, these are allowed to be buried on farm (https://www.gov.uk/guidance/fallen-stock). In addition, when there is a catastrophic epizootic, mass burial may be a necessary option, as it was during the foot-and-mouth disease outbreak in the UK in 2001 [22]. Rules issued by the United States Department of Agriculture (USDA) do advise burial as an option for fallen stock but also state that this process should be limited by a comprehensive list of factors such as soil depth and the need to prevent drainage into the water table and hence into drinking water (https://www.aphis.usda.gov/emergency_response/tools/on-site/htdocs/images/nahems_disposal.pdf). With regard to the UK, it is entirely likely that BSE-infected cattle carcasses remain decomposing in soil. To address possible concerns that BSE infectivity may disseminate from such burial sites, we have looked to model the burial and spread of BSE infectivity within field-scale experiments using animal bioassay as the primary read-out.

We have used rodent-passaged BSE (301V) as the BSE agent, as at the outset of this study it allowed known amounts of infectivity at high titre to be used and also had an accompanying mouse bioassay with relatively short incubation periods [23]. This rodent-passaged BSE has been shown to have very similar stability to cattle BSE, as measured by infectivity, under relatively low-stringency inactivation conditions, such as incubation with acid or SDS [31]. However, it shows less stability than cattle BSE under high-stringency conditions, for example, autoclaving or incubation with acid in combination with SDS [31]. The conditions in the present study are likely to cause relatively low levels of inactivation, and so 301V is likely to reflect cattle BSE stability. However, even if conditions are more favourable to prion inactivation than anticipated, the presence of 301V prion will still represent the survival of cattle BSE. Infected brain macerate was buried in large volumes of soil in lysimeters and placed either within the heads of cattle or as an unprotected bolus. Heads were buried for 5 years, and boluses for 4 years. We also tested the rainwater that eluted through glass fibre filters at the bottom of the lysimeters. Weather conditions included high amounts of rain and a range of temperatures (from – 11 °C in December 2010 to + 28 °C in July 2009).

These two experiments were designed to demonstrate what could happen to TSE infectivity when buried in near-natural conditions. There is an increasing amount of information on survival/degeneration of infectivity and/or PrP^Sc^ in brain homogenates mixed with various soils and therefore in full contact with soil particles and micro-organisms [32]. In the present study, brain macerates were used, and the tissue was buried in the form of undispersed boluses so that soil/micro-organism contact was at best around the outer edges of the bolus sample or, when inside the cows’ heads, not in immediate contact with the sample. Studies from other labs have also attempted to investigate the survival and/or destruction of TSE infectivity and PrP^Sc^ in simulated conditions which might occur in soil. For example, repeated cycles (n = 10) of drying and wetting resulted in reduction in detectable levels of PrP^Sc^ and infectivity from TME-affected hamster brain homogenates mixed with clay loam [33]. However, survival of infectivity may be different in the semi-protected conditions provided by being inside an animal carcass or in a solid bolus unmixed with soil. Here, we have shown that high levels of TSE infectivity can, and probably in most circumstances do, survive in brain tissue underground for very long periods of time – at least five years in this case – without significant loss of TSE infectivity. For example, we found that high levels of TSE infectivity were readily detected in cattle skull contents at similar levels each year for five years. Data also showed only limited migration from the site of deposition; infectivity was present in limited samples at 25 cm distance from the burial site, and up to 20 cm above the site and 50 cm below it. However, PrP^Sc^ was detectable by sPMCA in extracts of filters through which had drained a proportion of the water eluting from a 42.4-m^3^ clay lysimeter. PrP^Sc^-positive samples were found up to 25 months after the burial of the 301V bolus sample. These data confirmed limited bioassay data that found infectivity in a filter extract sample taken at 25 months, one of two samples analysed. These results suggest a risk of spread of infection into watercourses if burial sites are not contained properly.

Our studies did not test the wide variety of soil chemistries and environmental conditions that might be encountered by TSE infectivity when deposited into soil. Nevertheless, these results should be taken into account when considering the future use and possible remediation of sites where BSE infectivity has been deposited. It should be assumed that high levels of BSE remain even after many years.

## Electronic supplementary material

Below is the link to the electronic supplementary material.

Supplementary material 1 (DOCX 293 kb)
