# Supplementary material for: BSE infectivity survives burial for five years with only limited spread
Source: Arch Virol. 2019 Feb 24;164(4):1135–45. doi: 10.1007/s00705-019-04154-8 (PMC6420460; doi:10.1007/s00705-019-04154-8)
Supplement: Supplementary file 1 — Supplementary material 1 (DOCX 293 kb) [file 705_2019_4154_MOESM1_ESM.docx]

**Supplementary Information**

**Table S1: Physicochemical characteristics of the two soils**

| **Property** | **Clay Loam** | **Sandy Loam** |
| --- | --- | --- |
| Texture | Fine | Coarse |
| Pore space | Small | Large |
| Structure | Well developed | Not very developed |
| Water holding capacity | High | Low |
| Drainage | Slow | Rapid |
| Organic matter content | High | Low |
| Organic matter decomposition | Slow | Rapid |
| Capacity to hold nutrients | Large | Little |
| Nutrient supplying power | High | Low |
| Leaching of pollutants | Retards | Allows |
| Shrinkage/Swelling | Moderate to high | Little or none |
| Compaction | Easily compacted | Resists |
|  | | |
| pH (H_2_O) | 5.60 | 6.98 |
| *Particle size* | | |
| % clay (< 0.002 mm) | 6.4 | 23.0 |
| % silt (0.002 – 0.02 mm) | 11.9 | 13.8 |
| % sand (0.02 – 2,0 mm) | 81.7 | 63.2 |
| Texture classification | Sandy clay loam | Loamy sand |
| OC content, % | 2.44 | 0.49 |
| CEC, cmol_c_ kg^-1^ | 7.78 | 4.87 |
| *Exchangeable cations cmol_c_ kg^-1^* | | |
| Ca | 7.37 | 5.82 |
| Mg | 0.90 | 0.61 |
| Na | 0.096 | 0.089 |
| K | 0.166 | 0.122 |
| Fe | < 0.005 | < 0.005 |
| Mn | 0.029 | < 0.005 |
| Al | 0.11 | 0.035 |

All analyses (except particle size) of Scottish soils were carried out by INRA, France.

**Weather patterns October 2005 to March 2011**

The information on weather patterns throughout the period of the experiments provides context for comparing our experimental results with situations where contamination of the environment with TSE-infected material is suspected to have occurred.

The lysimeter site was at about 180 meters above sea level and situated to the south of Edinburgh in the East of Scotland, UK. The five years of the experiment had higher than average rainfall for the area, particularly in 2006, 2007 and 2008, which is reflected in our data (Figure S1A). On several occasions, sustained high levels of rainfall led to prolonged saturation of the soil in the lysimeters, particularly those with clay soil. Surface water tended to accumulate on the clay lysimeters and was pumped off on occasion. The monthly rainfall shows the variability in rainfall over periods of time, with little seasonal pattern. The site was also exposed to substantial snowfalls, particularly in the winter of 2010/11.

In general, the clay lysimeters retained higher soil moisture levels than the sandy soil lysimeters. The clay soil lysimeters remained saturated near the bottom of the soil column nearly all the time throughout the life of the experiments. Only the small sand lysimeters showed a reduction in the moisture levels at the 90 cm point before the next spell of rainy weather saturated the soil again. At higher levels in the lysimeters some reduction in moisture content was seen at times.

Monthly average air temperatures are given in Figure S1B. Four lysimeters (the two large lysimeters and two of the small lysimeters that contained the control bovine heads) were fitted with devices to continuously monitor temperature and moisture content at 3 depths: 30, 60 and 90 cm below the surface. The temperatures varied within the lysimeters in parallel with the air temperatures although tending to have attenuated extremes (data not shown).

**Figure S1:** Weather conditions at the lysimeter site 2005-2011. A: monthly rainfall (mm). B: monthly average air temperature (C). * date of cow head burials. # date of bolus burials.

**Figure S2:** Representation of the presence and location of 301V BSE infectivity in the small clay (A) and sand (B) lysimeters. Direction of sampling and the depth are shown, samples were taken 25 cm from the central core or at the central core. The black oval represents the location of the buried head containing the 301V brain macerate. Grey circles represent the location of infectivity that was present in any of the 5 lysimeters for each soil type that were sampled over the 5-year duration of the experiment.

**Figure S3. Rolling monthly rainfall compared with sPMCA analysis of water filters from the clay lysimeter draining line.** Rainfall on to the lysimeter site is shown as a rolling amount (mm, plotted for each day over a 48 month timescale) after burial of 301V infected bolus in the lysimeter containing clay soil. Positive and negative sPMCA results are indicated by filled and empty arrows respectively and are placed on the graph at the month of removal from the draining line over the 48 month period.
